# Effects of maximal-versus submaximal-intent resistance training on functional capacity and strength in community-dwelling older adults: a systematic review and meta-analysis

**DOI:** 10.1186/s13102-022-00526-x

**Published:** 2022-07-16

**Authors:** Liam T. Pearson, David G. Behm, Stuart Goodall, Rachel Mason, Samuel Stuart, Gill Barry

**Affiliations:** 1 Department of Sport, Exercise and Rehabilitation, Faculty of Health and Life Sciences, Northumbria University, Newcastle Upon Tyne, NE1 8ST UK; 2 School of Human Kinetics and Recreation, Memorial University of Newfoundland, St. John’s, Canada; 3 Northumbria Healthcare NHS Foundation Trust, Tyne and Wear, UK

**Keywords:** Resistance training, Ageing, Timed up and go, Sit to stand, SPPB

## Abstract

The objective of this systematic review is to investigate the effects of different methods of resistance training (RT) on functional capacity in older adults. A systematic literature search was conducted using PubMed, SPORTDiscus, Web of Science, CINAHL, Cochrane CENTRAL, ClinicalTrials.gov databases, from inception to December 2021. Eligibility criteria consisted of randomised control trials (RCT’s) involving maximal-intent resistance training (MIRT), where participants (aged 60+) had specific instruction to move *‘as fast as possible’* during the concentric phase of the exercise. Twelve studies were included within the meta-analysis. Divided into functional capacity and strength-related outcomes; Improvements were evident for timed-up-and-go (*p* = 0.001, SMD: − 1.74 [95% CI − 2.79, − 0.69]) and knee extension one-repetition maximum (1RM) (*p* = 0.01, SMD: − 1.21, [95% CI − 2.17, − 0.25]), both in favour of MIRT, as well as in 30 s sit-to-stand in favour of T-STR (*p* = 0.04, SMD: 3.10 [95% CI 0.07, 6.14]). No statistical significance was found for combined functional capacity outcomes (*p* = 0.17, SMD: − 0.84, [95% CI − 2.04, 0.37]), with near-significance observed in strength-related outcomes (*p* = 0.06. SMD: − 0.57, [95% CI − 1.16, 0.02]) favouring MIRT. Heterogeneity for FC-outcomes was observed as Tau^2^ = 4.83; Chi = 276.19, df = 14, I^2^ = 95%, and for strength-outcomes Tau^2^ = 1.290; Chi = 109.65, df = 115, I^2^ = 86%. Additionally, MIRT elicited substantial clinically meaningful improvements (CMI) in Short Physical Performance Battery (SPPB) scores but fell short of CMI in 400 m walk test by 0.6 s. In conclusion, this systematic review highlights the lack of sufficient and quality evidence for maximal- versus submaximal-intent resistance training on functional capacity and strength in community-dwelling older adults. Study limitations revolved around lack of research, low quality (“low” PEDro score), and largely due to the fact many comparison studies did not match their loads lifted (1500 kg vs. 500 kg), making comparisons not possible.

**Supplementary Information:**

The online version contains supplementary material available at 10.1186/s13102-022-00526-x.

## Introduction

The World Health Organisation (WHO) predicts by 2050 “the number of people aged 60 years or older will rise from 900 million to 2 billion” [1]; and with the decline in neuromuscular function and subsequent functional capacity (FC) associated with ageing [2–6], more investigation is required for improving FC and physical performance of older adults. The National Strength and Conditioning Association (NSCA) and The WHO World Confederation for Physical Therapy (WCPT) [7, 8], in 2019, both released position statements asserting the benefits of resistance training (RT) for older adults as *almost overwhelming*, and for the use of RT in older adults for maximising FC. Outcomes related to FC can be assessed using a standardised Short Physical Performance Battery (SPPB) test [9], which has shown to be applicable in: age-associated declines in muscle mass [4, 10], multiple geographical settings [11, 12], general and clinical populations [13–16], obese and frail adults [17], in Alzheimer’s patients with early-stage dementia [12], older adults with and without mobility limitations ([4]), older adults at risk of disability ([18]), and has shown potential as an early predictor of declining FC in only adults in clinical populations [19]. Resistance training, as recommended by WHO 2020 guidelines, has shown positive correlations in physical function in older adults [20–22], as well as enhancements in outcomes in SPPB scores, strength, gait speed, sit-to-stand, and reduction of hip pain [23–28]; All of which, are outcomes supported by the American College of Sports Medicine (ACSM) [29] and Physical Activity Guidelines Advisory Committee (PAGAC) [30] for functional parameters linked to improvements in FC. Less is known about how the intent of resistance training can impact FC and strength outcomes in older adults, due to the lack of articles directly comparing speed or intent of movement during exercise programs.

Resistance training can be defined as; the use of load, machinery, or Q2own body weight while exercising the muscles [31] or, a modality of exercise used to increase the body’s ability to overcome load [32]. RT is used as the main intervention within this review as there is overwhelming support for the use of RT in the prevention of sarcopenia [33–36], one of the most common conditions suffered by up to 13% of all older adults, and upwards of 50% in those aged 80 and above [37]. It is therefore suggested the stronger an older adult, the better they will likely cope with basic physical activity guidelines [20, 38, 39] and are therefore more likely to adhere to, or even exceed said guidelines, leading to an improved FC.

Evidence to support the use of high-velocity resistance training (HVRT) in non-athletic populations is beginning to emerge, with current papers from Bernat, Candow [40] and Englund, Sharp [41], both concluding HVRT in untrained ageing adults shows encouraging results compared to traditional strength training (T-STR). This supports research from 2008 which advocates maximal power and optimal velocity training in older women were found to improve physical performance, and be significant mobility factors in older adults [42]. Commonly with HVRT, the protocol is to have a slow and controlled eccentric action followed by a powerful concentric reaction, with eccentric action being linked to improvements in outcome measures in older adults well-being, mobility, survival, and activities for daily living [43]. T-STR is commonly accepted as moderate-load (60–80% 1RM), multi-joint RT between three to five sets of eight to twelve repetitions, with emphasis on slow and controlled repetitions and three minutes recovery between sets [44–46]. For this systematic review, and due to the fact older adults may not express high-velocities common in HVRT or velocity-based training (VBT) literature, it was therefore deemed more appropriate to investigate maximal-intent resistance training (MIRT), which can be defined as; the purposeful intention of the individual to attempt to move as fast as possible, regardless of the imposed resistance during RT, whether through intrinsic motivation or encouragement from an external source.

The nervous system is also an important contributor to mobility, or more specifically, mobility limitations, with said limitations typically observed through ageing [2, 47, 48], with evidence to suggest RT may be a possible solution to stall what we lose through ageing [49–51]. A specific ‘power’ RT intervention conducted by Rodriguez-Lopez, Alcazar [48], Reid, Martin [52], McKinnon, Connelly [5] also observed significantly improved neuromuscular activation in older adults, with similar findings of neuromuscular and cognitive performance observed by Marques, Neiva [53]. Due to the neural adaptations observed following RT, further investigation is needed regarding the potential significance and application across all general, ageing, and clinical populations [54–59]. Neuromuscular responses to RT do not have to be conducted at high velocities, there are links to the *intent* of movement being an equal factor to neural improvements in muscle activation and movement time [60], with evidence replicated over 10 years later [61]. Although there is merit to physical improvements in older adults that relate to FC from MIRT [5, 24, 25], the underpinning neural mechanisms of the observed neuromuscular changes remain unclear [62]. Observations also suggest links of psychology to the neuromuscular system [63]; with Behm [64] additionally observing links to high-velocity training adaptations may involve significant neural adaptations, including Ansdell, Škarabot [65] who observed differences in neural responses between sexes.

The objective of this systematic review is to investigate the effects of Maximal-intent resistance training (MIRT) versus traditional resistance training (T-SRT) on functional capacity and strength in older adults.

## Methods

A systematic literature search was conducted on PubMed, SPORTDiscus, Web of Science, CINAHL, Cochrane CENTRAL, ClinicalTrials.gov databases, from inception to December 2021, using standard operators (AND, OR). Guidelines from Preferred Reporting Items for Systematic Reviews and Meta-Analyses (PRISMA) [66] were followed throughout. Search terms used throughout each database were “resistance training” OR “strength training” AND “VBT” OR “velocity-based” OR “MVC” OR “MVIC” OR “RFD” OR “maximal-intent” OR “explosive” AND “older adult”. Combined results from all databases were screened by the lead author (LP) and one additional reviewer (RM), using the Rayyan web-based platform [67]. Any discrepancies in the results were reviewed by a third, blinded, reviewer (GB). Where necessary data was missing, attempts were made to contact the authors. The authors, title and year of publication, sample size, participant characteristics (age, sex, and health status), intervention characteristics (group/intervention, exercises, length of intervention, weekly frequency of training, training time per session), retention rates and adherence, outcome measures for each group at baseline, follow-up, and change scores (mean and standard deviation [SD]), were extracted manually by LTP using the online platform Rayyan [67]. If mean and standard error (SE) were reported, the SD was calculated from the SE using the following formula: SD = SE*√*n*, with *n* denoting sample size [68]. If age was only reported per group, the Cochrane calculator was used to transform age into a pooled mean and SD for the study characteristic table [68]. Where data was reported in Newtons (N), this was divided by 9.81 to equate to kilograms (kg).

### Statistical analysis

Software used for the meta-analysis of this systematic review were Review Manager (RevMan V5.3; Cochrane Collaboration, Oxford, UK) using continuous outcomes, change scores in mean and standard deviation (SD), and reported as standardised mean difference (SMD) using 95% confidence intervals. Between-study variability was examined for heterogeneity, using I^2^ statistics for quantifying consistency, with thresholds being set at I^2^ = ≤ 25% (low), I^2^ = 26–74% (moderate), and I^2^ =  ≥ 75% (high) [69]. For conservative reasons, a random-effects model of meta-analysis was applied to the combined data.

### Inclusion and exclusion criteria

Inclusion criteria followed Population, Intervention, Comparison, Outcome, and Study (PICOS) and Physiotherapy Evidence Database (PEDro) methodologies [66, 70] (Table1):

**Table 1:** PICOS inclusion and exclusion criteria for data synthesis

*Population*	
Conducted on community-dwelling older adults aged ≥ 60 years	
*Intervention*	
Concentric muscle action	
Randomised control trial	
Participants must have been instructed to move *“as fast as possible”* during the concentric phase, or instructions of a similar description	
*Comparison*	
Comparison between RT performed whilst being encouraged to concentrically move as fast as possible (MIRT) vs. slow-to-moderate velocity (T-STR)	
Studies that reported pre- and post-intervention scores for changes in SPPB score	
*Outcome*	
Primary outcome measure was SPPB score, or any individual test derived from the SPPB tests (30-s chair stand (STS), timed-up-and-go (TUG), or balance testing)	
Secondary outcomes were dynamic leg press 1RM and knee-extension 1RM, 400-m (400 m) walk, 6-min walk test	
*Study*	
A minimum four-week intervention	Between-group design
Published in a peer-reviewed journal	Full-text available in English
*Exclusion criteria*	
Did not specify whether maximal concentric velocity was encouraged	Used concurrent training methods
Did not reply with additional information upon request within 30 days	The article had been retracted
Supplementary/dietary combined intervention	A quasi-experimental research design

## Results

The meta-analysis comprised 12 studies totalling 371 participants. When combining related outcomes, no significant improvements were found for FC outcomes (*p* = 0.17, SMD: − 0.84, [95% CI − 2.04, 0.37]) (Fig. 2), however, near-significance with moderate magnitude of effect was observed in strength-related outcomes (*p* = 0.06. SMD: − 0.57, [95% CI  − 1.16, 0.02]), favouring MIRT (Fig. 3).

### Search results

**Fig. 1:**
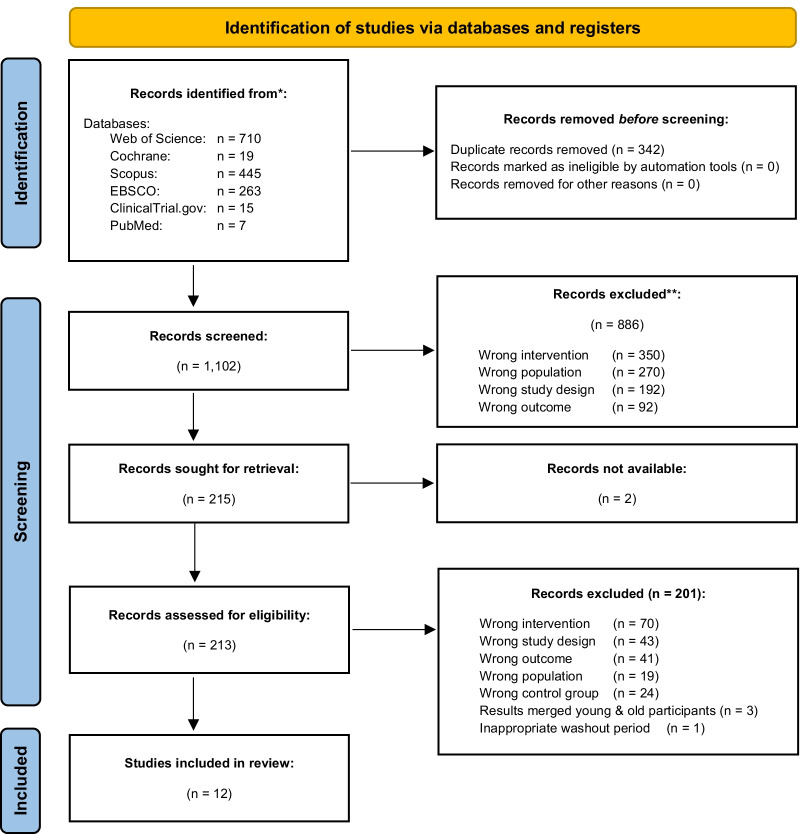
PRISMA flow diagram

Figure 1 below indicates the PRISMA flow diagram of search results.

### Intervention attributes

All studies focused primarily on lower-body RT, with zero focusing exclusively on the upper body. Across all studies, sample sizes ranged from 18 to 50. Seven studies (70%) [27, 71–76] mixed both male and female participants. Most studies recruited from links with community-dwelling adults and organisations [27, 71–77]. All participants came from the same cohort of recruitment, with a similar number of participants per group. A variety of common RT exercises and derivatives were utilised across all studies such as squats, leg press, lunges, knee flexion and extension.

Only one study provided a power calculation, Tiggemann, Dias [78]. Four studies (40%) [27, 73, 75, 76] recorded their method of randomisation, all using a computerised random number generator. Only two studies (20%) included information regarding safety of RT or injuries/illnesses sustained during the intervention; Gray, Powers [27] reported no injuries and suggested MIRT as safe and effective, and Miszko, Cress [72] reported a 22% (11/50 participants) drop-out through injuries and/or personal medical reasons. Three studies (30%) [27, 78, 79] failed to include dropouts, whilst one paper (10%), Drey, Zech [73], cited power training appeared more likely to influence dropout than T-STR. No participants were blinded to the intervention hypothesis, and no sham exercises or groups were implemented. No no-exercise control groups were included within this systematic review or meta-analysis.

### Risk of bias and methodological quality

The Physiotherapy Evidence Database (PEDro) scale [80], ranging from zero (‘poor’) to ten (‘excellent’), was used to assess risk of bias and methodological quality of studies included in quantitative synthesis [70]. PEDro scores were directly sourced from the PEDro database (https://search.pedro.org.au/search), revealing a mean score of 4.6 ± 1.1 points. All included studies adhered to randomised groups. All eligible studies and their respective PEDro scored can be found below (Table 2):

**Table 2:** PEDro scores of studies included in data synthesis

Reference	PEDro score	Score obtained from pedro database?
Richardson, Duncan [81]	7/10	Y
Coelho-Junior and Uchida [76]	6/10	Y
Drey, Zech [73]	6/10	Y
Henwood and Taaffe [71]	5/10	Y
Bottaro, Machado [82]	4/10	Y
Henwood, Riek [74]	4/10	Y
Lopes, Pereira [77]	4/10	Y
Lopes, Pereira [83]	4/10	Y
Marsh, Miller [75]	4/10	Y
Ramirez-Campillo, Castillo [79]	4/10	Y
Tiggemann, Dias [78]	4/10	Y
Miszko, Cress [72]	3/10	Y

As some studies reported age as a range, and not mean ± SD, it was not possible to calculate the overall mean age range for all studies included within this systematic review, only an age range between 60 and 90 years.

Training durations ranged from 6 to 48 weeks. Training frequency ranged from 2 to 6 days per week. Exercise sets were consistent throughout, with 2–3 sets for RT programmes and repetitions per exercise ranging from 8 to 14. Rest intervals ranged from 60 to 300 s. Exercise intensity ranged from 40 to 90% of one-repetition maximum (1RM).

With Henwood and Taaffe [71] comparing four different interventions; high-velocity, low-velocity, high-velocity with gymnastics, and no training; only data from high-velocity and low-velocity training groups were extracted for comparison.

A breakdown of study characteristics can be found in the Additional file 1 alongside this manuscript, titled Additional file 1: Appendix A.

### Sensitivity analysis

The removal of eight low-quality studies (PEDro score of 0–4) resulted in no change for the overall effect of MIRT on FC-related outcomes (*p* = 0.16, SMD: -0.83 [95%CI -2.00, 0.34], I^2^ = 92%), however, the effect of MIRT on strength-related outcomes would be deemed significant (*p* = 0.006, SMD: − 1.39 [95% CI − 2.38, − 0.41], I^2^ = 88%). Sub-group analysis was not possible for 400 m walk or SPPB, due to the lack of studies in the revised pool (one).

For outcome measures relevant to this review, clinically meaningful improvements are estimated as being minimal at 0.3–0.8 points of change and substantial at 0.8–1.5 points of change for SPPB scores, and 20–30 s reduction in time for the 400 m walk test [84].

### Functional capacity outcomes

See Fig. 2.

**Fig. 2:**
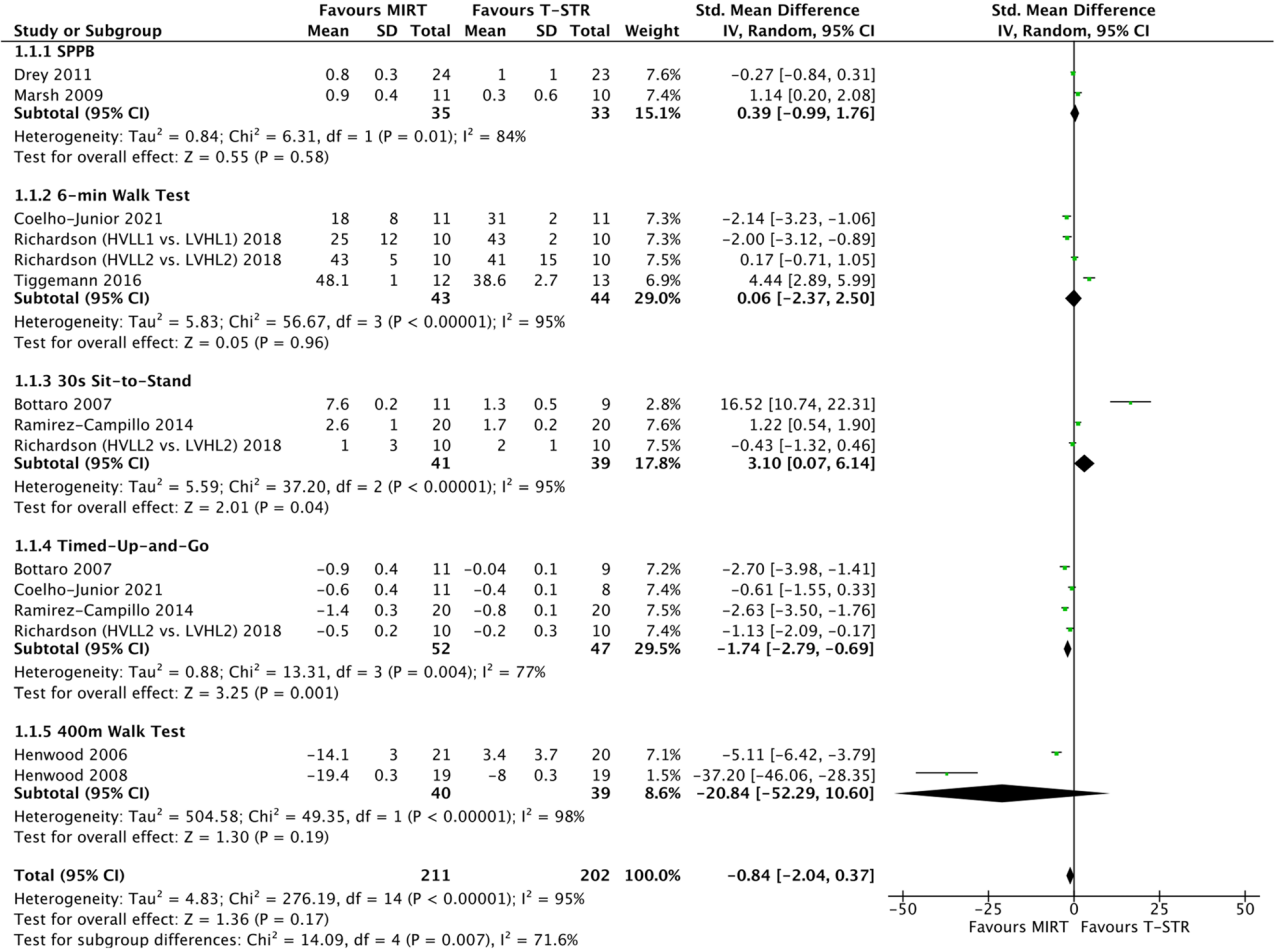
Standardised mean difference (95% CI) from baseline of the effect of maximal-intent training on functional capacity outcomes

#### Strength outcomes

See Fig. 3.

**Fig. 3:**
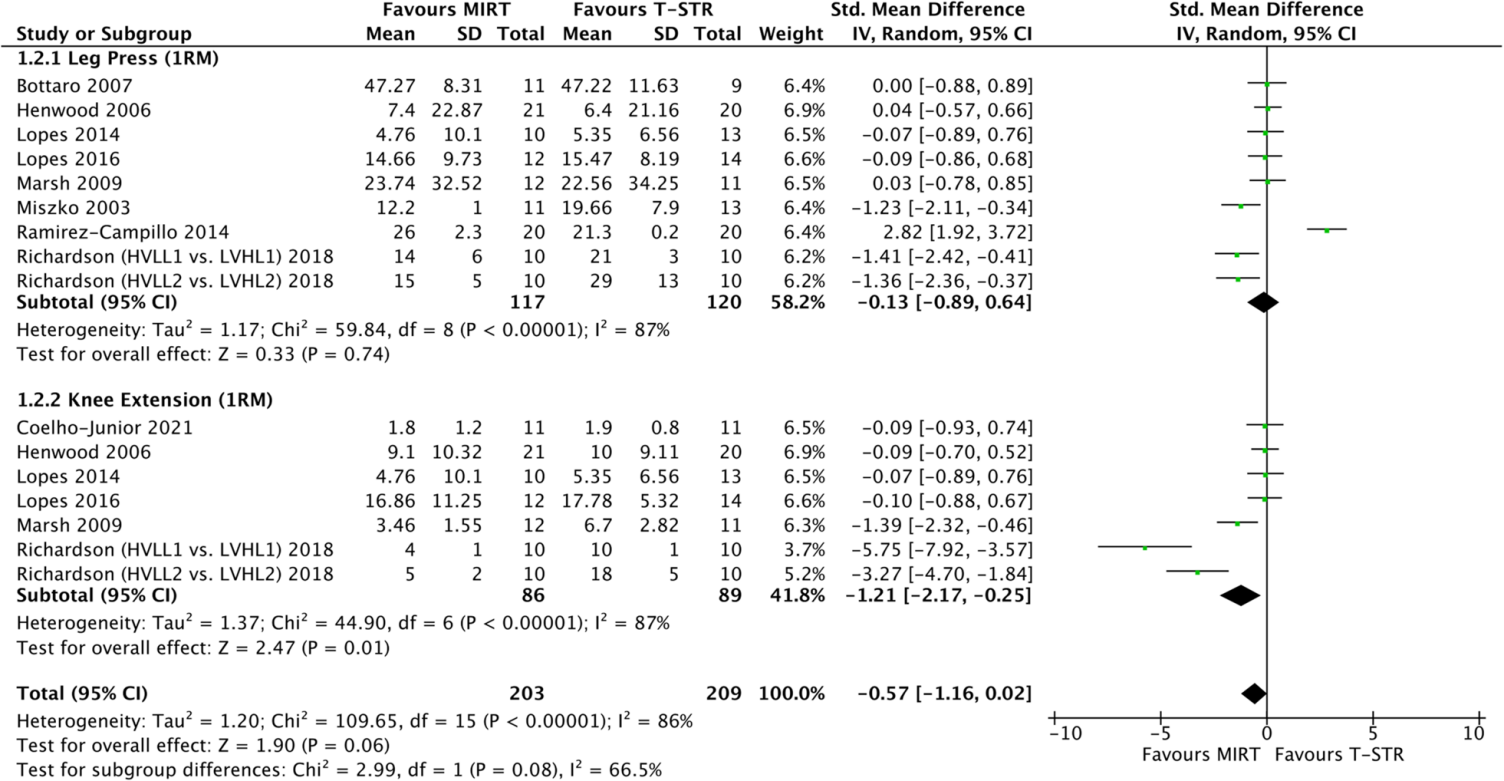
Standardised mean difference (95% CI) from baseline of the effect of maximal-intent training on strength outcomes

##### SPPB scores

No statistical significance was reported with the two studies (68 participants) for changes in SPPB scores (*p* = 0.58, SMD: 0.39 [95% CI − 0.99, 1.76], I^2^ = 84%). Substantial clinical improvements were observed in favour of MIRT, with change scores reported as 0.9 and 0.8.

##### Leg press 1RM

No statistical significance was found with the nine studies (237 participants) for changes in leg press 1RM (*p* = 0.74, SMD: − 0.13 [95% CI − 0.89, 0.64], I^2^ = 87%).

##### Knee extension 1RM

Statistical significance was found to favour MIRT in the seven studies (175 participants) for improvements in knee extension 1RM, (*p* = 0.01, SMD: − 1.21 [95% CI − 2.17, − 0.25]), I^2^ = 87%).

##### 30 s sit-to-stand (STS)

Analysis of 30 s STS initially included five studies, of which only three (amounting to 80 participants) were able to be included due to two studies providing no change scores. Statistical significance was found for improvements in 30 s sit-to-stand scores, favouring T-STR (*p* = 0.04, SMD: 3.10 [95%CI 0.07, 6.14], I^2^ = 94%).

##### Timed-up-and-go (TUG)

Analysis of TUG included six studies, of which only four studies (99 participants) were able to be included due to two studies providing no change scores. Statistical significance was found for improvements in TUG scores, favouring MIRT (*p* = 0.001, SMD: − 1.74 [95% CI − 2.79, − 0.69], I^2^ = 77%).

##### 400m walk

There was no statistical significance with the two studies amounting (79 participants) investigating 400 m walking scores (*p* = 0.19, SMD: − 20.84 [95% CI − 52.29, 10.60]), I^2^ = 98%). MIRT fell short of clinically meaningful difference by 0.6 s, with T-STR being over 10 s adrift.

##### 6-minute walk test (6MWT)

Statistical significance was severely lacking with the four studies (87 participants) for improvements in 6MWT scores (*p* = 0.96, SMD: 0.06 [95% CI − 2.37, 2.50]), I^2^ = 95%).

## Discussion

This systematic review and meta-analysis examined the effect of maximal-intent RT vs traditional RT on functional capacity and strength outcomes. These findings suggest no statistically significant differences between different training on aggregated functional capacity or strength outcomes. However, subgroup analysis demonstrated statistically significant improvements on timed-up-and-go, 30 s sit-to-stand scores, and knee extension 1RM, favouring MIRT. This systematic review and meta-analysis are a first of its kind to the author(s) knowledge, encompassing 12 studies. Whilst some studies provide statistical significance, others show low quality (“low” rating via PEDro score) and high heterogeneity (I^2^ of 95% for FC and 85% for strength) within additional subgroup analysis, meaning this review cannot draw meaningful conclusions on the effects of MIRT on FC in older adults. Research in this area requires further investigation.

Sensitivity analysis would suggest that no study included within this review overestimated the effect of MIRT on FC, however, statistically significant changes were observed when on strength outcomes when lower quality studies were removed, suggesting lower quality studies overestimate the effect of MIRT on strength in community-dwelling older adults.

Additionally, a meta-analysis was performed and included on outcomes with only one or two articles due to the fact that FC outcomes are a combination of multiple factors/tests, and therefore every included paper has significance in the overall outcome of FC in community-dwelling older adults.

### Impact of MIRT on functional capacity

Although ACSM [29], PAGAC [30], NSCA [7], The World Confederation for Physical Therapy [8], and The Journal of Geriatric Nursing [85] all support the use of MIRT for older adults, this investigation only found statistically significant improvements in timed-up-and-go, 30 s sit-to-stand scores, and knee extension 1RM. No significance was found in SPPB, 6MWT, 400 m walk or leg press 1RM. Clinically meaningful improvements were observed in SPPB scores [84].

During screening, it was noted when standardised tests were selected such as the SPPB or walking tests, researchers were modifying these standardised tests, thus quashing any form of comparison. This must be addressed in future research if we are to gain further insight into the most effective methods of collecting and collaborating data on improving functional capacity in older adults. An agreed upon, and regularly undertaken, Functional capacity testing programme is needed to aid comparisons between interventions. The findings of some of the subgroup analyses such as 6MWT, 400 m walk, TUG, and STS should be considered with caution due to the small-to-moderate effect sizes and low participant numbers leading to potential underpowered studies skewing results. Low number of studies eligible for data synthesis, including low-to-moderate quality and bias assessment by means of PEDro scores are also reasons to interpret this meta-analysis with care. There also needs to be more consistency and symmetry in the designing of studies, for example, many studies conducted velocity-based interventions but did not standardise the exercises conducted within each group, leading to a differing specificity and training-effect between groups. Titles of research also need considerations, such as the use of velocity-based that were not using velocity to modify intra-sets or -repetitions, thus were not velocity-based and would be better categorised as velocity-monitored.

From papers captured within this review, there are suggestions of minimum dose of interventions being inadequate, with Bottaro, Machado [82] citing older adults require a higher dose of weekly RT, suggesting a minimum of three training sessions per week for 16 weeks as a maintenance dose for neuromuscular adaptation, and only one day per week for young adults.

Time-under-tension (TUT), which would commonly be used in a T-STR block, improved muscle strength specifically in older adults (*p* < 0.01), with larger effects being seen for those who were under tension the longest (maximum 6 s) [86]. There was also a relatively novel finding within Borde, Hortobagyi [86]’s research, in that, although they advocated for one less weekly training sessions per week than Bottaro, Machado [82], they found that a rest of 4 s per *repetition* was found to be most successful for improvements in RT-related outcomes in older adults. Giving a 4 s rest per repetition also heavily favours MIRT, as this recovery between repetitions may allow for greater amount of high-velocity repetitions, therefore improving the number of repetitions to allow for neuromuscular adaptations [82], whilst also obtaining the benefits of MIRT [7, 8, 85]. Rest per repetition was not recorded in any of the studies in this review, thus highlighting a key area for further investigation.

Functional capacity is a complex outcome measure with many contributing factors, such as both lower and upper body strength, mobility, and diet. Functional capacity is also subject to psychological and psychosocial considerations. More robust RCT’s are necessary to enhance future meta-analyses, investigating the magnitude and direction of effect across different dosages, and velocities, of exercise on FC outcomes in older adults. The outcome measures in most of the studies in the review are relatively subjective in nature and only tell us about one global functional outcome (i.e., walking speed). Future research could use wearable technology (i.e., wearable sensors) to assess subtle changes in movement for a number of outcome measures including 6MWT, TUG, Sit-to-stand, and 400 m walking. Allowing for more robust outcome measures to be captured and potentially more information regarding FC.

### Outcome measures

Practitioners also need to ensure they are using the abbreviation 6MWT appropriately, as there are many studies confusing 6MWT with both 6-min walking test, and 6-m walking test, again, a standardised set of these practises and abbreviations would be of benefit to the scientific and wider community.

### Plausible mechanisms

Whilst the actual mechanisms responsible for improvements are yet to be distinguished, most aspects of FC revolve around knee and hip dominant movement patterns, such as the ability to stand up from a chair, locomotion, and stair climbing, all preferably pain-free and without superfluous fatigue [87]. It is therefore expected that exercises such as the leg press and knee extension (of which at least one was present in all interventions) all improved aspects of FC, theorised as these exercises closely mimic the movement patterns of FC outcomes such as STS and TUG. These theories were observed in the results of this systematic review, and likewise as observed by McKinnon, Connelly [5] who found as older adults age, their reliance shifts from ankle-, to knee- and hip-dominance during locomotion. As previously noted, it is suggested that greater velocities produce greater neural adaptations [61], therefore MIRT could illicit similar adaptations due to participants being asked to move as fast as possible, resulting in surpassing of thresholds for type II fibres that may elicit neural adaptations [88]. MIRT may also improve firing frequencies within the muscle, and since rate of force development is associated with higher firing frequencies and increase in muscle tension, this may be why we see results suggesting MIRT improving FC such STS, TUG, and leg press, as all are movement patterns that require high levels of force and require high levels of action potential to execute [5, 48]). There is also speculation that the greater forces thought to be sustained within the musculotendinous unit due to the higher movement speed than that of T-STR and TUT, and lack of central circulatory stress observed through higher volume training, may be resulting in peripheral muscle adaptation [89].

### Clinical interpretations

No statistically significant improvements were found for SPPB, 6MWT, 400 m walk or leg press 1RM. However, improvements were found in favour of MIRT for knee extension 1RM and TUG, as well as 30 s sit-to-stand in favour of T-STR; These findings align with research by Bean, Kiely [90], who’s investigation highlighted significant associations between improvements in leg power (regardless of one’s strength) and clinically meaningful improvements in FC in older adults. Highlighting the need for further investigation as to whether the associations between leg power are, for example, ankle, knee, or hip dominant, which could lead to significantly isolated and specific exercise regimes recommendations for older adults looking to improve their FC.

### Study limitations

Many additional studies were eligible for this meta-analysis based on the inclusion criteria, but workloads between groups were not comparable, for example groups lifting a total of 522 kg versus 1500 kg (across all sets and repetitions). This must be addressed in future research. Exercise groups *must* begin to be matched on volume and intensity to allow for comparisons of intervention. The average of the included studies was 4.6/10 (Table 2). All included studies were conducted over differing periods of time, utilising differing training prescriptions.

## Conclusion

In conclusion, this systematic review highlights the lack of sufficient and quality evidence for maximal-versus submaximal-intent resistance training on functional capacity and strength in community-dwelling older adults. No statistical significance was present in combined FC outcomes (*p* = 0.17), but MIRT observed near-significance for improvements in strength (*p* = 0.06). Clinically meaningful improvements were observed in SPPB scores, showing potential for MIRT over T-STR resistance training recommendations if further research continues to support these findings. Further investigation is necessary to observe whether similar clinically meaningful improvements are replicated, in hopes of providing future guidelines for MIRT in older adults for both physiological and neurological adaptations over T-STR; Rest-between-repetitions has also been highlighted as significant interest as a direction for future investigations. Due to the less time-consuming and lesser short-term and long-term fatiguing nature of MIRT over T-STR, MIRT has a greater chance of being adopted by the community-dwelling older adult community.

## Supplementary Information

**Additional file 1.** Appendix A.

## Data Availability

The datasets used and/or analysed during the current study is available from the corresponding author on reasonable request.

## Abbreviations

ACSM: American College of Sports Medicine
CMI: Clinically meaningful improvements
CI: Confidence interval
CIDESD: Research centre in sports sciences, health sciences and human development
COPD: Chronic obstructive pulmonary disease
ECC: Eccentric
ES: Effect size
FC: Functional capacity
HVRT: High-velocity resistance training
LVRT: Low-velocity resistance training
MIRT: Maximal-intent resistance training
NHS: National Health Service (UK)
NSCA: National Strength and Conditioning Association
PAGAC: Physical Activity Guidelines Advisory Committee
PEDro: Physiotherapy evidence database
PICOS: Population, intervention, comparison, outcomes and study design
PT: Power training
PWV: Pulse-wave velocity
QoL: Quality of life
RCT: Randomised control trial
RT: Resistance training
SD: Standard deviation
SE: Standard error
SPPB: Short physical performance battery
STS: Sit-to-stand
T- SRT: Traditional strength training
VBT: Velocity-based training
WHO: World Health Organisation
MVIC: Maximal-intent voluntary contraction
ESPEN: European Society for Clinical Nutrition and Metabolism
